# Bacterial flagellar motility on hydrated rough surfaces controlled by aqueous film thickness and connectedness

**DOI:** 10.1038/srep19409

**Published:** 2016-01-13

**Authors:** Robin Tecon, Dani Or

**Affiliations:** 1 Soil & Terrestrial Environmental Physics, Department of Environmental Systems Science, ETH Zürich, Universitätstrasse 16, 8092 Zürich, Switzerland

## Abstract

Recent studies have shown that rates of bacterial dispersion in soils are controlled by hydration conditions that define size and connectivity of the retained aqueous phase. Despite the ecological implications of such constraints, microscale observations of this phenomenon remain scarce. Here, we quantified aqueous film characteristics and bacterial flagellated motility in response to systematic variations in microhydrological conditions on porous ceramic surfaces that mimic unsaturated soils. We directly measured aqueous film thickness and documented its microscale heterogeneity. Flagellar motility was controlled by surface hydration conditions, as cell velocity decreased and dispersion practically ceased at water potentials exceeding –2 kPa (resulting in thinner and disconnected liquid films). The fragmentation of aquatic habitats was delineated indirectly through bacterial dispersal distances within connected aqueous clusters. We documented bacterial dispersal radii ranging from 100 to 10 μm as the water potential varied from 0 to –7 kPa, respectively. The observed decrease of flagellated velocity and dispersal ranges at lower matric potentials were in good agreement with mechanistic model predictions. Hydration-restricted habitats thus play significant role in bacterial motility and dispersal, which has potentially important impact on soil microbial ecology and diversity.

Dispersal and migration are fundamental ecological processes that allow organisms to explore new habitable sites, to avoid adverse local conditions and to reduce competition^1^. The same principles apply to microscopic organisms such as bacteria, which represent the most abundant forms of life on Earth^2^. Bacterial cells can disperse in liquid environments via Brownian motion and passive diffusion, but this has been shown to be an inefficient mode of locomotion for nutrient interception^3^, hence, most bacterial species have evolved means of active locomotion^4^. Notably, mechanisms of self-locomotion include swimming powered by rotating appendages (flagella)^5^,^6^,^7^. Flagellated bacteria typically rely on gradient-guided swimming (chemotaxis) to orientate in a heterogeneous aqueous habitat^8^,^9^,^10^. However, many natural habitats are not water-saturated and this has important ecological consequences for bacterial motility and dispersal. In soils, which harbor high bacterial density and diversity^11^, flagellar motility is physically constrained by the size and fragmentation of aqueous habitats and their connectedness by micrometric aqueous films^12^,^13^. Therefore, flagellar motility in unsaturated soils is restricted to relatively rare and short-lasting wetting events (e.g., rainfall)^12^ in which swimming is an opportunity to explore and colonize new surfaces.

In natural porous media such as soils, water is retained in pores and on solid surfaces by matric forces (capillarity and adsorption). The lower energy state of this water (relative to bulk water under similar conditions) is represented by the matric potential (*ψ*_*m*_), which is expressed as energy per unit volume of water (i.e., a pressure) and is always subatmospheric (negative)^14^,^15^. Consequently, as soil dries the matric potential becomes more negative (lower) with *ψ*_*m*_ of zero indicating complete saturation^14^. Hence, low values of matric potential in soil result in thin and disconnected aqueous films^16^,^17^ and thus hinder microbial rates of motion and dispersal. Recently, Dechesne *et al*.^18^ have demonstrated that bacterial flagellar motility on hydrated porous surfaces is controlled by surface water matric potential. To do so, they have measured bacterial growth and motility on a porous ceramic surface model (PSM) that mimics conditions found in unsaturated soils and that allows direct surface observation^19^. In this experimental system, aqueous film thickness on the porous surface is function of surface roughness and matric potential (which is set by a controlled suction applied to the liquid medium in the PSM). As the aqueous film becomes thinner (due to lower *ψ*_*m*_ values), the velocity of swimming cells is reduced by increased viscous drag (due to interactions with the surface) and capillary forces. Importantly, Dechesne *et al*. have shown that flagellar motility ceases to play a significant role in dispersal when matric potential is lowered to –2 kPa (i.e., conditions drier than *ψ*_*m*_ = 0 kPa)^18^,^19^. This small range (0 to –2 kPa) for motility on hydrated surfaces supports the view that bacterial swimming is globally limited in unsaturated soils^13^,^20^,^21^, in which typically water is retained at *ψ*_*m*_ values much lower than –2 kPa. Dechesne *et al*. have predicted the effective aqueous film on the PSM to be thinner than 1.5 μm at –2 kPa, but this has not been confirmed experimentally. In addition to reducing liquid film thickness (and hence swimming velocity) on surfaces, low (negative) matric potential values are associated with reduced aqueous phase content and connectivity that result in formation of spatially isolated ‘micropools’ connected only by micrometric liquid films. These phenomena and their implications for bacterial cell velocity and dispersal are further illustrated in Supplementary Fig. S1 online.

In the present work, we address the twofold effect of aqueous film thickness on bacterial cell velocity and aqueous habitat fragmentation. We have used a 3D laser scanning microscope to study in detail surface and liquid film on a porous surface similar to the one used by Dechesne *et al*. We characterized the porous ceramic surface at a microscopic level that was not accessible in previous studies, and we provided for the first time direct measurement of aqueous film thickness on rough surfaces. We measured the joint effects of surface geometry and aqueous configuration on the flagellar motility of the soil bacterium *Pseudomonas protegens* (a rhizosphere bacterium often used as a model organism in soil microbial ecology^22^,^23^,^24^). We limited our investigations to the type of flagellar motility known as bacterial swimming; other types of locomotion such as bacterial swarming rely on *en masse* cell movement (often on agar surfaces)^7^ and were not investigated in this study. Our primary objectives were to relate individual bacterial cell velocity with aqueous film thickness, and to use bacteria as living gauges to measure the fragmentation of the aqueous habitat at the microscale. We measured and linked together these bacterial dispersal metrics, and we demonstrated that they agreed well with theoretical predictions. Aqueous phase connectivity between habitats (which we define as connectedness) is of great significance for microbial interactions, diversity and dispersal in natural porous environments such as wet soils^25^,^26^,^27^. By coupling microhydrological conditions with flagellar motility and dispersion distances, our study aims to link behaviour of individual microorganisms with physical microenvironments and to provide mechanistic basis for emergent community level diversity and organization.

## Results

### Variation of aqueous film configuration and thickness with matric potential

**Figure 1:**
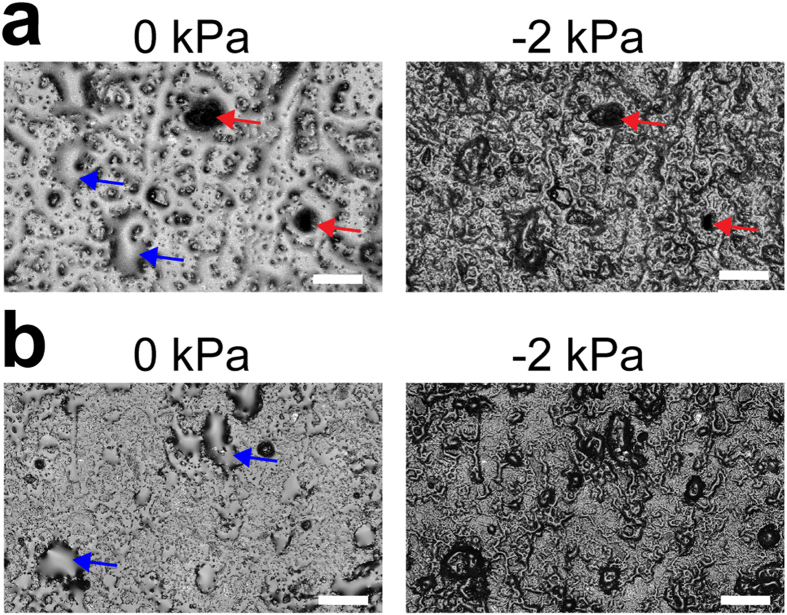
Aqueous film configuration on the ceramic Porous Surface Model (PSM). Micrographs depict a sample area of a ‘rough’ (**a**) or ‘smooth’ (**b**) ceramic PSM surface at matric potentials of 0 kPa and –2 kPa, obtained by laser scanning microscopy (bar is 200 μm). The ‘smooth’ surface was obtained by grinding a ‘rough’ (untreated) surface with a polishing disc to remove irregularities. Under nearly saturated conditions (0 kPa) the surface is covered by a thick aqueous film that locally forms connected micropools (blue arrows). Under a mild suction (–2 kPa) the micropools drain and the film thickness on the PSM is reduced, revealing the natural roughness (red arrows mark high spots on the ‘rough’ ceramic PSM).

**Figure 2:**
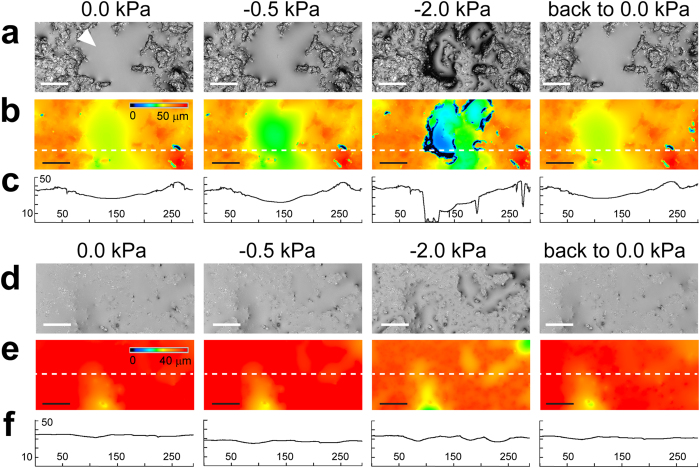
Dynamics of aqueous film configuration on PSM. Changes of hydration conditions were applied sequentially. Images show an exemplary area of ‘rough’ PSM (**a–c**) and of ‘smooth’ PSM (**d–f**). (**a,d**) Micrographs of PSM surface obtained by laser scanning microscopy. Aqueous film appears as a smooth, homogeneous surface (white arrowhead), distinct from the rough ceramic surface. Bar is 50 μm. (**b,e**) Heatmaps of the areas showing the relative height position in z-axis (blue indicates lowest positions, red highest positions). Bar is 50 μm. (**c,f**) xz-graph showing the surface profile of a PSM transect (axes values are in μm). The transect y position is indicated by the white dashed line in (**b,e**).

**Figure 3:**
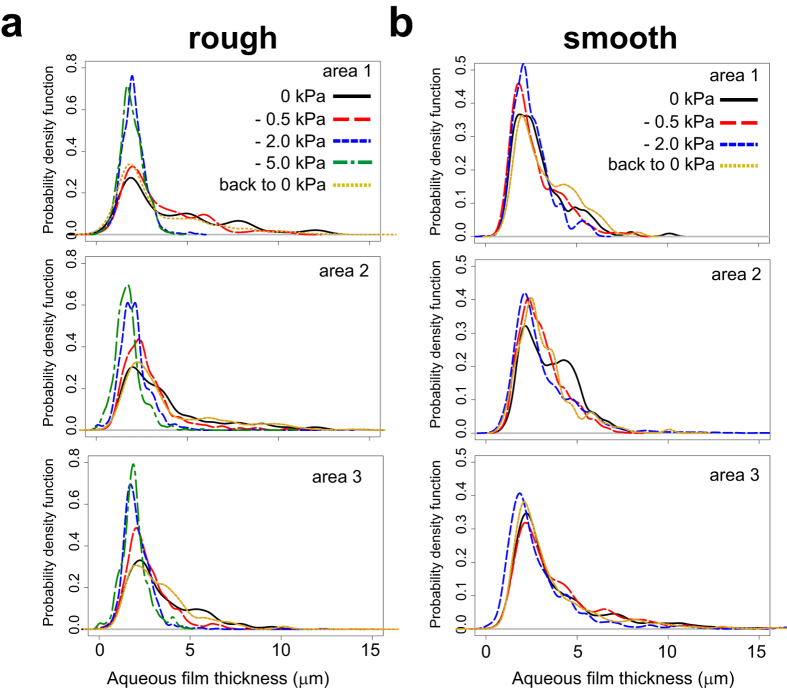
Measurement of aqueous film thickness on PSM at different matric potentials. Aqueous films were measured on the surface of ‘rough’ (**a**) and ‘smooth’ (**b**) PSM, using 3D scanning laser microscopy, with a detection limit of ≈2 μm. Three random PSM areas of 200 μm x 289 μm were analyzed. For each area, aqueous film data from ten equidistant x-axis transects were pooled, and the probability function was estimated using kernel density estimation. The measurements at different matric potential values were done sequentially.

We developed a new ceramic-based porous surface model (PSM) similar to the set-up described in Dechesne *et al*.^19^ (see Methods and Supplementary Fig. S2 online). This set-up permits systematic variations of liquid film configuration on the hydrated porous surface while allowing for direct observation of bacterial cells. A 3D laser scanning microscopy (3D LSM) provided direct characterization of aqueous film configuration and thickness on PSM having two different surface roughness characteristics (‘smooth’ and ‘rough’, see Supplementary Fig. S2). All surfaces used in this study were porous and rough, hence the resulting aqueous films were much thicker than nanometer-scale adsorbed films on flat solid surfaces^12^,^17^ that do not support bacterial flagellated swimming. We observed that, even for a water-saturated surface (i.e., matric potential is zero), the aqueous film configuration was heterogeneous at the microscale, as illustrated by the presence of extensive micropools connected by thin liquid films (Fig. 1). In our experimental system, lower matric potential values were obtained by lowering the position of the reservoir bottle relative to the position of the ceramic surface (see Methods for calculation details). On the PSM, a matric potential of –2 kPa drained liquid from the porous surface, resulting in visibly drier conditions (Fig. 1). We further investigated the dynamics of hydration changes by systematically varying the surface matric potential on the PSM. Our measurements followed a temporal sequence: starting from 0 kPa, we gradually lowered *ψ*_*m*_ (–0.5 kPa, –2.0 kPa, *etc*.) to modify the aqueous film configuration and we finally brought the system back to 0 kPa (Fig. 2). Lowering the matric potential to –0.5 kPa had only moderate effects on the aqueous film configuration, whereas –2 kPa and lower values (drier surface) drastically modified surface films, resulting in complete disappearance of surface micropools and emergence of pronounced surface roughness that previously was covered by thick liquid films (particularly on rough PSM). The reestablishment of initial matric potential conditions (0 kPa) largely restored initial aqueous film configuration (Fig. 2). Liquid drainage from the PSM occurred within a few seconds after lowering the relative position of the control liquid reservoir. We observed a certain hysteresis (i.e., a discrepancy between sorption and desorption processes) in the PSM response, manifested by slower rates of rewetting relative to drainage rates following step change in matric potential values. Supplementary Video S1 shows the dynamics of aqueous configuration in a sampling area as *ψ*_*m*_ was varied from 0 to –10 kPa, and then brought back to 0 kPa. The 3D LSM measurements of aqueous film thickness distribution at randomly selected observational areas of the PSM consistently produced a right-skewed probability function (Fig. 3). The foregoing analysis, in agreement with visual observations (Fig. 2), confirmed heterogeneity of the aqueous phase configuration primarily on the rough PSM as related to surface geometry and complex liquid contact lines. Aqueous films thicker than 5 μm were relatively rare and receded as matric potential decreased, practically disappearing at –2 kPa. Quantification of the subsequent restoration of the aqueous film to its initial configuration showed that it was not as complete as suggested by visual observations. Overall, the effects of lowering matric potential were more pronounced with ‘rough’ than with ‘smooth’ PSM (Fig. 3).

### Aqueous film configuration controls bacterial motility and dispersal rates

We used the soil bacterium *Pseudomonas protegens* to measure swimming motility on the PSM. *P. protegens* is a rod-shaped bacterium that uses flagella located at one of the cell’s poles for motility. We obtained, under the selected growth conditions, a population that consisted almost entirely of swimming cells. To evaluate how swimming behaviour was affected by variations in hydration conditions, we transferred the swimming *P. protegens* bacteria to the PSM. Then, we observed flagellar motility in individual cells while the aqueous film configuration was sequentially varied from water-saturated (*ψ*_*m*_ = 0) to unsaturated conditions (*ψ*_*m*_ = –0.5 kPa, –2 kPa, *etc*.), and finally brought back to initial conditions (*ψ*_*m*_ = 0). After each change of matric potential, we waited ~5 min before measuring cell motility.

**Figure 4:**
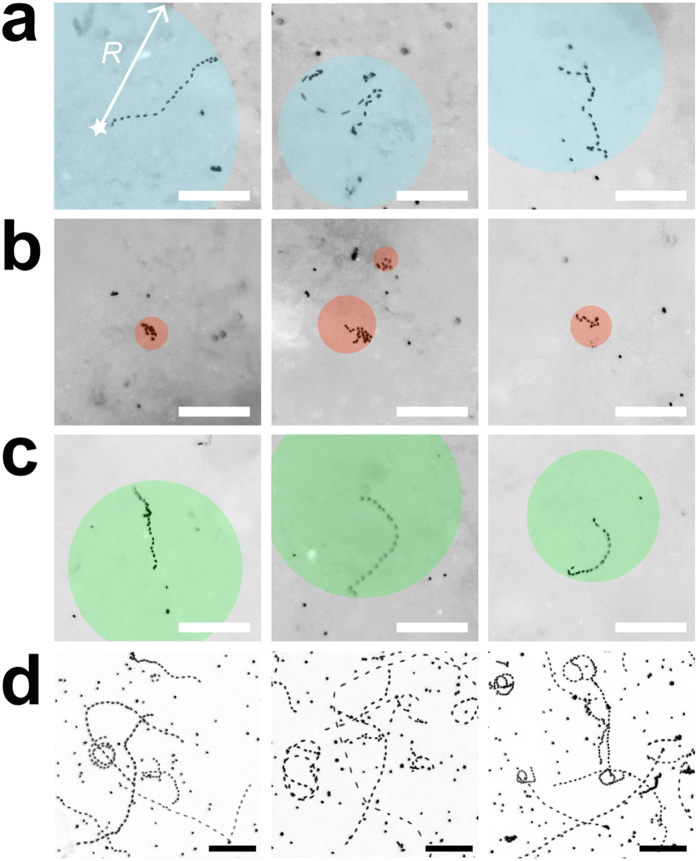
*Pseudomonas protegens* swimming trajectories in aqueous films. Micrographs show the projection of a stack of 36 sequential fluorescent images recorded during a 10-sec time lapse. Bacteria were stained with Syto9 for visualization by epifluorescence microscopy. Swimming trajectories appear as dotted lines, with inter-dot gaps proportional to cell velocity. Bar is 50 μm. Examples of individual bacteria swimming on ‘rough’ PSM are shown (**a–c**). Cells that appear as a single dot are immobilized (attached to the surface). Coloured circles indicate the maximal dispersal distance, defined by radius *R* measured from the cell position at time 0 (star). (**a**) Bacteria swimming at matric potential of 0 kPa (water-saturated conditions). (**b**) Swimming trajectories are constrained when matric potential is brought to –2 kPa (drier conditions). (**c**) Swimming is reinstated when the matric potential is brought back to 0 kPa. Note that bacteria shown in (**a**–**c**) are not the same. (**d**) Control of bacteria swimming between a glass slide and coverslip. Bacteria that do no exhibit swimming trajectories are attached to glass.

An important observation was that the majority of bacterial cells (>90%) became immobilized on the PSM, irrespective of the hydration conditions. This is expected given the preponderance of thin (<5 μm) liquid films on PSM even under saturated conditions (Fig. 3). We therefore focused our analysis on the subset of cells that were not attached or pinned on the surface. In this subset, swimming trajectories ranged from straight lines to circles when no suction was applied (*ψ*_*m*_ = 0) (Fig. 4a, Supplementary Video S2). The rates and ranges of flagellar motility were practically unaffected when *ψ*_*m*_ was set to –0.5 kPa, but were already limited at –2 kPa, as shown by slower and tightly (spatially) constrained trajectories (Fig. 4b, Supplementary Video S3). At the end of the hydration sequence we reinstated the initial conditions, which effectively restored initial swimming characteristics (Fig. 4c). We observed *P. protegens* swimming in a liquid film between two glass slides as a control representing bulk liquid (Fig. 4d). Cell swimming trajectories in the control were less constrained than trajectories on the PSM (even under saturated conditions), which highlighted the effects of surface roughness and geometry on bacterial motility. Altogether, these direct observations provided new insights into the dynamics and reversibility of the conditions experienced by microorganisms in their aqueous habitat.

**Figure 5:**
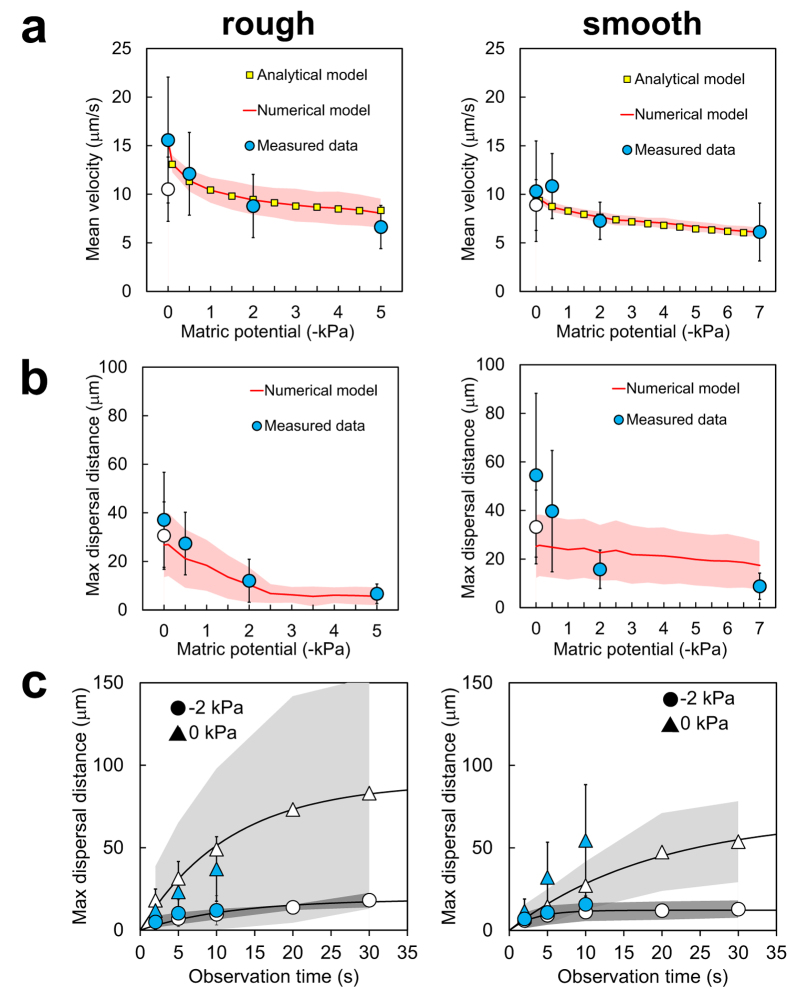
Quantification of *Pseudomonas protegens* flagellar motility on PSM and comparison with model simulations. We tested PSM with either ‘rough’ (left panels) or ‘smooth’ (righ panels) surface characteristics. (**a**) Mean swimming velocity, which is the average of calculated swimming speeds >3 μm/s (the method detection limit). (**b,c**) Maximal dispersal distance, which is the radius of a circle centered at the initial cell position at time 0 and reaching to the most distant position occupied by the cell during the observation time (see Fig. 4a). (**a,b**) Circles show average values calculated from *n* independent swimming trajectories (observation time is 10 s): Rough: *n* = 20 (0 kPa), *n* = 20 (–0.5 kPa), *n* = 17 (–2 kPa), *n* = 15 (–5 kPa), *n* = 21 (back to 0 kPa); Smooth: *n* = 19 (0 kPa), *n* = 19 (–0.5 kPa), *n* = 17 (–2 kPa), *n* = 20 (–7 kPa), *n* = 24 (back to 0 kPa). Error bars are 1 standard deviation (SD). Matric potential was sequentially lowered (0 kPa, –0.5 kPa, *etc.*) and finally brought back to 0 kPa (indicated by an open circle). We used an analytical model for flagellar motility that takes into account the hydration-dependent resistive forces acting on a cell (see Methods). With numerical simulations, shaded area is ±1 SD calculated from 100 realizations. (**c**) Effects of observation time on the measured maximal dispersal distance at 0 kPa and –2 kPa. Blue (closed) symbols show 10 s data used in (**b**) and additional values for 2 s and 5 s observations. Error bars are ±1 SD. Open symbols show results from replicate experiments with observation time extended to 30 s. Rough: *n* = 23 (0 kPa), *n* = 4 (–2 kPa); smooth: *n* = 16 (0 kPa), *n* = 12 (–2 kPa). Shaded areas are ±1 SD.

We further quantified bacterial swimming motility based on a set of indicators that were previously used to assess single-cell motility on similar PSM^18^. These indicators included the mean swimming speed, the maximal dispersal distance and the mean fraction of motile time in individual motile cells (Fig. 5 and Supplementary Fig. S3 online). Results clearly illustrated that bacterial swimming velocity was reduced, the distances traversed by individual cells were shorter, and the swimming periods were fewer with decreasing matric potential (i.e., with drier conditions and fragmentation of aqueous habitats on the surface). These trends were similar for both ‘rough’ and ‘smooth’ porous surfaces (Fig. 5a,b). The decrease in flagellar motility was reasonably well described by analytical (for cell velocity) and numerical models that were previously validated^18^ (Fig. 5a,b). In the experiments, returning to initial hydration conditions (0 kPa) restored flagellar motility to nearly its initial levels, which demonstrated how physical conditions control microbial behaviour. However, bacterial swimming in the system was not completely reversible. The maximal dispersal distances on PSM were lower after the hydration sequence (although the difference was only significant on ‘smooth’ PSM, with a p-value of 0.008 calculated with a two-tailed t-test). Similarly, the average cell velocity after *ψ*_*m*_ was reinstated to 0 kPa was also reduced compared to initial values (p-value of 0.002 on ‘rough’ PSM). Average cell velocities were clearly lower on the PSM than in the control glass slide (see Supplementary Fig. S4 online), due to the physical constraints exerted on individual cells swimming on hydrated rough surfaces. The distribution of cell velocities also differed between the control slide experiments for ‘rough’ and ‘smooth’, which could also reflect variations between *P. protegens* cultures in independent experiments. It is important to note that the control of flagellar motility between glass slides was performed after the end of the hydration sequence on PSM, using the same suspension of stained cells that was transferred to the PSM. This demonstrated that cell behaviour on PSM could not be explained by prolonged exposure to growth medium or fluorescent dye, and that the bacterial cells were physiologically able to swim throughout the experiment.

An important result not previously quantified is the size of connected aquatic domains on the porous surface (Fig. 5b,c and Supplementary Fig. S3), and is measured by the maximal dispersion radii of swimming cells on the hydrated surface at a given matric potential (Fig. 4). Given the experimental setup, these measurements should be interpreted as relative measures of *ψ*_*m*_ dependency that vary with the duration of the recorded swimming period. In our experiments, which were routinely based on 10 s of observation time, we showed that dispersal distances and data variations decreased with decreasing matric potential (Fig. 5b). We increased the observation time to 30 s to further investigate the limits of dispersion (Fig. 5c), and we found that dispersal distances at –2 kPa rapidly reached a plateau in the range 10–20 μm , while dispersion at 0 kPa continued to increase with prolonged observation time. This indicated that at low matric potentials (≤–2 kPa) bacterial dispersal distance was not a strong function of observation time, and measured dispersal radius effectively gauged the size of the connected aquatic cluster accessible by the bacterium, which was 5–10 μm at the lowest matric potential we measured (–7 kPa, see Supplementary Fig. S3). We normalized these radii values by the maximal dispersal distance observed in an unconstrained environment (between glass slides) to represent the aqueous phase fragmentation of the hydrated porous surface (Supplementary Fig. S3), and the observations were in agreement with trends predicted by percolation theory. These results confirmed the rapid decrease in connectedness below a certain matric potential threshold (–2 to –5 kPa)^20^.

We examined whether our observations were species-specific by measuring the flagellar motility of *Escherichia coli* on rough PSM. *E. coli* is a rod-shaped bacterium originating from the human gut that sometimes contaminates soil environments, and that swims by flagella distributed around its entire cell body. The cell preparation had to be amended with an additional culture step, because *E. coli*’s swimming motility peaks in post-exponential growth phase but fades in stationary phase^28^. This modification of the experimental protocol yielded a population of *E. coli* with a high proportion of swimming cells, hence allowing comparison with *P. protegens*. Interestingly, results showed both departure from and confirmation of our findings obtained with the *Pseudomonas* species (Supplementary Fig. S5). Measuring *E. coli* flagellar motility on PSM proved very limited, as a very small proportion of cells were freely swimming. It was clear, however, that setting matric potential to –2 kPa and lower practically suppressed cell dispersal, similar to experiments with *P. protegens*. It was not clear if restoring initial conditions reinstated motility, as only few instances of bacterial swimming were observed (not enough to permit definitive analysis). These limitations to flagellar motility were due to conditions on the PSM, since *E. coli* cells swam freely in a control experiment between two glass slides (Supplementary Fig. S5).

## Discussion

This study quantified the roles of aqueous film thickness and connectedness in facilitating or suppressing bacterial motility and dispersal on surfaces mimicking unsaturated soils (PSM)^18^,^19^. Measurements using 3D laser scanning microscopy enabled us to document for the first time details of water configuration on the PSM consisting of isolated ‘micropools’ connected by thin liquid films (Figs 1, 2, 3), which match closely our conceptualized view of hydrated surfaces in unsaturated soil^13^ (Supplementary Fig. S1). As predicted theoretically, thick liquid films (>5 μm) were rare on the PSM and rapidly drained even under mild suction conditions (*ψ*_*m*_ ≤−2 kPa) (Figs 1 and 2). Our results also illustrated the importance of surface geometry, as the effects of drier conditions were accentuated on rougher surfaces. Surface roughness (geometry) shapes aqueous film thickness on the hydrated surface^16^ and hence plays a significant role in water configuration. In addition, we highlighted the aqueous configuration dynamics on the PSM, and showed both reversibility and hysteresis as drainage and wetting were applied (Fig. 2 and Supplementary Video S1). Overall, our results further support the use of the PSM as a realistic experimental model system for mimicking hydrated soil surfaces while allowing for direct observations of living bacterial cells.

We further linked the aqueous film configuration on the PSM to flagellar motility using the soil bacterium *Pseudomonas protegens*. We designed our experiments to observe specifically individual cell self-locomotion in liquid films (i.e., swimming motility^6^,^7^). We did not observe swarming motility, which is a collective form of surface motion^7^,^29^,^30^, typically induced on semi-solid and nutrient-rich agar media. Although swarming usually requires flagellar motility, it represents a distinct phenomenon from swimming^7^,^31^ and could potentially be investigated in future experiments on the PSM. In the present study, *P. protegens* swimming and dispersal unambiguously decreased with decreasing matric potential (Figs 4, 5), and almost ceased at *ψ*_*m*_ ≤–2 kPa. The reduction in *P. protegens* swimming velocity due to the thinning of the aqueous film matched well the predictions of a flagellar motility model that integrates the hydration-dependent resistive forces acting on a swimming microbial cell (Fig. 5)^13^. This further confirmed that flagellar motility is strongly controlled by water configuration on porous surfaces. It is important to distinguish the decrease in motility due to liquid-film thinning to other mechanisms of inhibition. For instance, various chemical compounds inhibit swimming by blocking the sodium or proton gradient required for flagella rotation in bacteria^32^,^33^,^34^. In addition, it has been shown that high hydrostatic pressure (80 MPa) physically blocks swimming motility in *E. coli*^35^. In our study, the inhibition mechanisms are different as the pressures applied (in the form of matric potential) are orders of magnitude lower than 80 MPa. Instead, on hydrated porous surfaces matric potential inhibits flagellar motility via its effect on water configuration at the microscale. The mechanism is twofold: (1) it gives rise to capillary pinning forces that overwhelm the propulsion force needed for bacterial swimming in bulk liquid, hence reducing cell velocity^13^,^18^, and (2) it reduces the aqueous phase connectedness of the system (two aqueous areas are ‘connected’ in the sense that a bacterium can travel from one to the other through the liquid film), hence limiting bacterial exploration range and dispersal^20^ (Figs 4 and 5 and Supplementary Fig. S1). In our experiments, thinner aqueous films lead to reduction in cell dispersal distances (radii) as function of the matric potential (Fig. 5). These results emphasize changes in aqueous film connectedness: the aqueous habitat becomes rapidly fragmented even at mild matric potentials, resulting in spatial isolation of the bacterial microhabitats. Our measurements of converging dispersal radii under low matric potentials document a phenomenon that was until now mostly theoretical^20^,^21^ (Fig. 5c and Supplementary Fig. S3). Our results also support the view that, in unsaturated porous media like soil, active dispersal by flagellar motility is restricted to a narrow range of matric potentials (a few kPa below zero)^20^. This supports the notion that the aqueous environment for bacterial life in soil (irrespective of type or geographical location) is highly fragmented and universally selects for sessile adaptations in microorganisms. In summary, the surface matric potential controls both thickness of the aqueous film and fragmentation of the aqueous habitat. The former has direct observable effects on flagellar motility, but we argue that the latter is far more important for microbial ecology: habitat fragmentation creates spatial microniches that are needed to explain the formation and maintenance of the enormous microbial diversity found in terrestrial environments.

Hydration levels in soil may vary rapidly due to drainage, evaporation or plant root uptake. Our study represents the first direct observation of the dynamic response of motile bacteria to fast changes in hydration conditions, hence mimicking the immediate effects of drainage and wetting on bacterial dispersal in soil. We sequentially changed the matric potential applied to the PSM (draining then rewetting, with transitions lasting for a few minutes), and we showed that bacterial ability to swim was correlated to this sequence (Figs 4 and 5). The results convincingly demonstrated that under these conditions bacterial motile behaviour was controlled by physical, and not physiological, factors: a population of temporarily immobilized bacteria was able to resume swimming as the aqueous configuration became again conducive to dispersal. This does not mean that physical and physiological factors are entirely independent. For instance, it is known that physically blocking flagellar rotation can activate the expression of regulatory pathways involved in long-term surface attachment, while repressing motility^36^. Recently, it was shown that the soil bacterium *Bacillus subtilis* used its flagella as ‘mechanosensors’ to trigger a signaling cascade leading to biofilm formation^37^, and it is expected that similar mechanisms exist in most motile bacteria. Such a drastic change in behaviour (from planktonic to long-term sessile multicellular life), however, takes hours or even days to unfold, and goes beyond the scope of our study. Nevertheless, in the future the PSM could prove a useful tool to address such behavioural programs.

Overall, these new findings are in agreement with the previous observations of Dechesne *et al*. with the bacterium *Pseudomonas putida*^18^. *P. putida* and *P. protegens* are phylogenetically related organisms that were isolated from the same habitat (soil). Both swim by polar flagella (lophotrichous flagellation), although the number of flagella varies between one and three for *P. protegens*^24^ and between five and seven for *P. putida*^38^. Interestingly, a similar matric potential threshold was observed with *Escherichia coli*, which is also rod-shaped but has flagella spread over its cell body (peritrichous flagellation). Although *E. coli* was isolated from the human gut and is therefore adapted to this particular environment, it can also be found in soil, where the survival and dispersal of pathogenic strains are a health concern^39^. These findings and previous results thus support certain broad generalizations including a matric potential dispersal threshold of ≈–2 kPa for motile rod-shaped bacteria^21^,^40^,^41^, but species idiosyncrasies should not be overlooked. We note that *E. coli*, which is the most studied bacterium with regards to flagellar motility and chemotaxis^42^, is a poor model for studies on porous surfaces. In summary, physics (through geometry and water potential) defines the global framework for unicellular motility on hydrated surfaces, but morphology and flagellation of microbial species play also a role.

In conclusion, our study of unsaturated porous surfaces revealed heterogeneous aqueous film configuration at the scale relevant to microorganism life. Bacteria were constrained by a fragmented aquatic habitat, in which active dispersion was limited to progressively smaller connected aqueous clusters with drier conditions. Additionally, bacterial cells that were swimming in bulk liquid became immobilized on the porous surface within thin aqueous films due to the action of viscous and pinning forces. These results illustrate the complexity of microbial ecology in soil, where heterogeneity in pore scale and water availability create myriad of microhabitats that directly control bacterial dispersal and activity. This is necessary insight for a better understanding of the ‘soil-microbe’ system, to use the term coined by Young and Crawford^43^, and this stresses the importance of studying soil microorganisms at the most critical scale for their functioning—the microscale.

## Methods

### Bacterial strains and culture conditions

*Pseudomonas protegens* CHA0 (formerly *P. fluorescens*) is a Gram-negative soil bacterium that possesses polar flagella^24^. *Escherischia coli* MG1655 is a Gram-negative ‘wildtype’ laboratory strain derived from the gut bacterium *E. coli* K-12^44^, and has peritrichous flagella. CHA0 and MG1655 were routinely grown in Lysogeny Broth (LB) liquid culture at 30 °C with shaking at 280 rpm, or on LB agar plates at 30 °C. The minimal medium M9^45^ supplemented with 0.4% glucose and 0.2% casamino acids (M9GCA) was used as a medium for swimming experiments.

### Preparation of the porous surface model (PSM)

The PSM was adapted from a previously published set-up^19^ and is described in Supplementary Fig. S2 online. It consisted of a porous ceramic disc of 1.4 cm diameter, cut from a 1-bar porous plate (Soilmoisture Equipment Corp., Santa Barbara, USA) with surface pore sizes smaller than 1 μm in radius. To produce a smoother surface than provided by the manufacturer, the ceramic discs were ground using a 20-μm polishing disc (3M, Rüschlikon, Switzerland) that reduced surface irregularities (the untreated and treated surfaces are respectively referred to as ‘rough’ and ‘smooth’ in this study). The ceramic disc was inserted into a PVC holder equipped with an O-ring to maintain the system airtight. Prior to experimentation, the PSM was autoclaved (121 °C, 1 bar) for 20 min. Then immersed in sterile M9GCA and placed in a vacuum chamber for at least 30 min to remove any trapped air bubble. The PSM was connected to a 250 ml-bottle containing 200 ml of M9GCA via a 100-cm Heidelberger extension line (B. Braun, Melsungen, Germany). For various steps of the experiment, we applied a predefined suction to the system (to control the effective aqueous film thickness) by lowering the free liquid medium surface in the reservoir relative to the ceramic surface. This suction mimics the control of hydration conditions exerted by the soil matric potential (*ψ*_*m*_), following a method commonly used in soil physics^46^. The height of the liquid column between the ceramic surface and the liquid medium surface in the reservoir determines the resulting suction applied to the PSM. The exerted matric potential is simply given by the relation *ψ*_*m*_ = ρ*gh*, with ρ the density of water, *g* the acceleration of gravity and *h* the height of the liquid column^46^. For more details on the PSM design, see the original publication of Dechesne *et al*. (2008)^19^.

### Scanning of PSM and measurement of aqueous film thickness

Surface roughness and aqueous film thickness analyses of PSM were performed with a Keyence VK-X200 3D laser scanning microscope (Keyence International, Mechelen, Belgium) using their VK Viewer acquisition software. Prior to analysis, all scanned data were processed using the ‘heightcut’ tool of the VK Analyzer software to reduce noise from the laser signal. Surface roughness of dry PSMs (‘rough’ or ‘smooth’) was characterized for 12 individual scanned areas using a 10× objective. To directly measure aqueous film thickness as a function of matric potential, we first saturated the PSM with deionized water, then the relative position of the reservoir was varied to establish a sequence of matric potential values (0, –0.5, –2, *etc*., and finally back to 0 kPa). We measured three randomly chosen areas using a 50X objective and the ‘transparent film’ acquisition mode of VK Viewer. Briefly, this measurement technique allows determination of the air-water and the water-solid positions along a chosen x-axis transect. The aqueous film thickness (in μm) is obtained by subtracting the bottom z-value from the top z-value at each pixel along the x-axis transect, wherever possible (the bottom film measurement is often discontinuous, as the signal may be lost due to surface geometry, and therefore we discard data at positions lacking a bottom z-value). According to the manufacturer, the measurement limit with a 50X objective is ≈2 μm. For each measured area, we pooled data from ten equidistant x-axis transects (covering the entire image) and calculated a probability density function for film thickness using the statistical software R (www.r-project.org) with the package ‘sm’.

### Observation and quantification of bacterial motility on PSM

An overnight LB culture of *P. protegens* CHA0 was diluted ten times in phosphate buffer saline (PBS). This dilution was mixed with the fluorescent dye Syto9 (Molecular Probes, Thermo Fisher Scientific, Waltham, USA) at a final concentration of 5 μM, and incubated in the dark for at least 30 min. After incubation, the suspension of stained cells was diluted in PBS in order to obtain an optical density at 600 nm (OD_600_) of ~0.005, and 20 μl of this dilution were pipetted evenly on the PSM. The position of the bottle was lowered relatively to the PSM in order to apply a gentle suction and absorb the droplet. Once the droplet was gone, matric potential was set to 0 and the system was let to rest for 5 min. Bacteria were visualized on the PSM with a DM6000 epifluorescence microscope (Leica Microsystems, Heerbrugg, Switzerland) using a 10×/0.3 HC PL Fluotar objective (Leica Microsystems) in combination with a L5 filter cube (exciter: 480/40; emitter: 527/30: beamsplitter: 505) (Leica Microsystems) and 100 ms of exposure time. Bacterial swimming was recorded in five areas of the PSM where swimming was taking place, using a sequence of 36 images taken during a 10-sec time lapse. The reservoir bottle was then lowered to set a new value of matric potential and the system was left to equilibrate for 5 min prior to repeating the acquisition of image series. The procedure was repeated for lower matric potential values (more negative), and finally the initial conditions (0 kPa) were reinstated for a final measurement. To specifically assess the maximal dispersal distance as function of observation time, we repeated the experiment using a sequence of 105 images taken during a 30-sec time lapse. The protocol for *E. coli* MG1655 preparation was slightly modified as follows. We inoculated 20 ml of fresh LB medium in a glass flask with 1/200 of overnight culture, and incubated it at 30 °C with shaking for 4.5 hours (OD_600_ was ~2.0). This step was necessary to induce swimming motility in *E. coli*. The culture was diluted an OD_600_ of ~0.5 in M9GCA (as PBS appeared to negatively impact *E. coli* swimming). Syto9 was added at a final concentration of 5 μM, and the suspension was incubated 10 min in the dark. Bacterial cells were diluted 50 times in M9GCA (OD_600_ of ~0.01). Twenty μl of this dilution were pipetted on the PSM, and the procedure was repeated as described above for CHA0. Stacks of sequential images were exported with the Leica LAS AF Lite software (Leica Microsystems) as avi files. We used ImageJ (www.imagej.nih.gov) to measure the motility metrics of individual swimming cells from image series as follows. We used ImageJ to convert the avi files as a stack of grayscale images in tiff format, to invert black and white colours and to adjust brightness and contrast to optimize cell detection. We used the ImageJ plugin ‘manual tracking’ to analyze individual swimming trajectories. The experimental speed detection limit with this image analysis technique was 3 μm/s. Probability density functions of swimming velocities were calculated using the statistical software R (www.r-project.org) with the package ‘sm’. To visualize swimming cell trajectories, we created a projection from the stack of images using the Image Stacks function ‘Z project’ in ImageJ.

### Modeling

The numerical and analytical models of flagellar motility and aqueous fragmentation on rough surfaces used in this study were described in details in previous publications^13^,^20^,^21^. Briefly, bacterial cell velocity in aqueous film is expressed as 
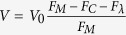
, with *V*_*0*_ the maximum average swimming velocity, *F*_*M*_ the propulsive force, *F*_*C*_the capillary pinning force and *F*_λ_ the resistive force that a swimming cell experiences. Both *F*_*C*_and *F*_λ_ hinder cell motility with decreasing effective aqueous film thickness on the surface. The aqueous film thickness varies as function of *ψ*_*m*_ and surface geometry (depth and spanning angle of idealized rough elements). Numerical simulations are based on a model 2D rough surface consisting of a network of simple elements (angular channels of various geometries) forming a hexagonal lattice, and populated with motile individual agents that grow and divide based on local conditions (representing motile bacterial cells). The distribution of channel angles was modified to account for the difference between ‘rough’ and ‘smooth’ surfaces. Different networks were generated for repeated simulations. A uniform chemotactic gradient was modelled (no preferred direction of swim). The hydration level and aqueous phase connectivity on the model rough surface varies with *ψ*_*m*_ and geometry. Channels are deemed connected when the aqueous film retained in these channels was thick enough to permit flagellar motility. Fragmentation of the aqueous network into disconnected clusters is triggered by lowering *ψ*_*m.*_

## Additional Information

**How to cite this article**: Tecon, R. and Or, D. Bacterial flagellar motility on hydrated rough surfaces controlled by aqueous film thickness and connectedness. *Sci. Rep.* **6**, 19409; doi: 10.1038/srep19409 (2016).

## Supplementary Material

Supplementary Information

Supplementary Video S1

Supplementary Video S2

Supplementary Video S3

## References

[b1] Begon M., Harper J. L. & Townsend C. R. Ecology: Individuals, Populations and Communities 3rd edn (Blackwell Science, 1996).

[b2] Whitman W. B., Coleman D. C. & Wiebe W. J. Prokaryotes: The unseen majority. P. Natl. Acad. Sci. USA 95, 6578–6583 (1998). 10.1073/pnas.95.12.6578 PMC33863 9618454

[b3] Purcell E. M. Life at low Reynolds number. Am. J. Phys 45, 3–11 (1977).

[b4] Dusenbery D. B. Living at Micro Scale: the Unexpected Physics of Being Small. (Harvard University Press, 2009).

[b5] Harshey R. M. Bacterial motility on a surface: many ways to a common goal. Annu. Rev. Microbiol. 57, 249–273 (2003). 14527279 10.1146/annurev.micro.57.030502.091014

[b6] Jarrell K. F. & McBride M. J. The surprisingly diverse ways that prokaryotes move. Nat. Rev. Microbiol. 6, 466–476 (2008). 18461074 10.1038/nrmicro1900

[b7] Kearns D. B. A field guide to bacterial swarming motility. Nat. Rev. Microbiol. 8, 634–644 (2010). 20694026 10.1038/nrmicro2405 PMC3135019

[b8] Adler J. Chemotaxis in bacteria. Annu. Rev. Biochem. 44, 341–356 (1975). 1094913 10.1146/annurev.bi.44.070175.002013

[b9] Fenchel T. Microbial behavior in a heterogeneous world. Science 296, 1068–1071 (2002). 12004118 10.1126/science.1070118

[b10] Stocker R. & Seymour J. R. Ecology and physics of bacterial chemotaxis in the ocean. Microbiol. Mol. Biol. R. 76, 792–812 (2012). 10.1128/MMBR.00029-12 PMC3510523 23204367

[b11] Torsvik V., Øvreås L. & Thingstad T. F. Prokaryotic diversity-Magnitude, dynamics, and controlling factors. Science 296, 1064–1066 (2002). 12004116 10.1126/science.1071698

[b12] Or D., Smets B. F., Wraith J. M., Dechesne A. & Friedman S. P. Physical constraints affecting bacterial habitats and activity in unsaturated porous media – a review. Adv. Water Resour. 30, 1505–152 (2007).

[b13] Wang G. & Or D. Aqueous films limit bacterial cell motility and colony expansion on partially saturated rough surfaces. Environ. Microbiol. 12, 1363–1373 (2010). 20192969 10.1111/j.1462-2920.2010.02180.x

[b14] Papendick R. & Campbell G. Theory and measurement of water potential In Water Potential Relations in Soil Microbiology (eds Parr J. F. . .), 1–22 (Soil Science Society of America, 1981).

[b15] Tuller M. & Or D. Retention of water in soil and the soil water characteristic curve In Encyclopedia of Soils in the Environment, Vol 4 (ed. Hillel D.), 278–289 (Elsevier, 2005).

[b16] Or D. & Tuller M. Flow in unsaturated fractured porous media: Hydraulic conductivity of rough surfaces. Water Resour. Res. 36, 1165–1177 (2000).

[b17] Tuller M. & Or D. Water films and scaling of soil characteristic curves at low water contents. Water Resour. Res. 41, W09403 (2005).

[b18] Dechesne A., Wang G., Gülez G., Or D. & Smets B. F. Hydration-controlled bacterial motility and dispersal on surfaces. P. Natl. Acad. Sci. USA. 107, 14369–14372 (2010). 10.1073/pnas.1008392107 PMC2922541 20660312

[b19] Dechesne A., Or D., Gülez G. & Smets B. F. The porous surface model, a novel experimental system for online quantitative observation of microbial processes under unsaturated conditions. Appl. Environ. Microb. 74, 5195–5200 (2008). 10.1128/AEM.00313-08 PMC2519293 18586968

[b20] Wang G. & Or D. A hydration-based biophysical index for the onset of soil microbial coexistence. Sci. Rep. 2, 881 (2012). 23181190 10.1038/srep00881 PMC3504331

[b21] Ebrahimi A. N. & Or D. Microbial dispersal in unsaturated porous media: characteristics of motile bacterial cell motions in unsaturated angular pore networks. Water Resour. Res. 50, 7406–7429 (2014).

[b22] Ueda A. & Saneoka H. Characterization of the ability to form biofilms by plant-associated *Pseudomonas* species. Curr. Microbiol. 70, 506–513 (2015). 25487118 10.1007/s00284-014-0749-7

[b23] Mascher F., Hase C., Bouffaud M.-L., Défago G. & Moënne-Loccoz Y. Cell culturability of *Pseudomonas protegens* CHA0 depends on soil pH. FEMS Microbiol. Ecol. 87, 441–450 (2014). 24224494 10.1111/1574-6941.12234

[b24] Ramette A. . *Pseudomonas protegens* sp. nov., widespread plant-protecting bacteria producing the biocontrol compounds 2,4-diacetylphloroglucinol and pyoluteorin. Syst. Appl. Microbiol. 34, 180–188 (2011). 21392918 10.1016/j.syapm.2010.10.005

[b25] Zhou J. . Spatial and resource factors influencing high microbial diversity in soil. Appl. Environ. Microb. 68, 326–334 (2002). 10.1128/AEM.68.1.326-334.2002 PMC126564 11772642

[b26] Carson J. K. . Low pore connectivity increases bacterial diversity in soil. Appl. Environ. Microb. 76, 3936–3942 (2010). 10.1128/AEM.03085-09 PMC2893478 20418420

[b27] Vos M., Wolf A. B., Jennings S. J. & Kowalchuk G. A. Micro-scale determinants of bacterial diversity in soil. FEMS Microbiol. Rev. 37, 936–954 (2013). 23550883 10.1111/1574-6976.12023

[b28] Amsler C. D., Cho M. & Matsumura P. Multiple factors underlying the maximum motility of *Escherichia coli* as cultures enter post-exponential growth. J. Bacteriol. 175, 6238–6244 (1993). 8407796 10.1128/jb.175.19.6238-6244.1993 PMC206719

[b29] Darnton N. C., Turner L., Rojevsky S. & Berg H. C. Dynamics of bacterial swarming. Biophys. J. 98, 2082–2090 (2010). 20483315 10.1016/j.bpj.2010.01.053 PMC2872219

[b30] Wu Y., Hosu B. G. & Berg H. C. Microbubbles reveal chiral fluid flows in bacterial swarms. P. Natl. Acad. Sci. USA 108, 4147–4151 (2011). 10.1073/pnas.1016693108 PMC3053958 21300887

[b31] Deforet M., van Ditmarsch D., Carmona-Fontaine C. & Xavier J. B. Hyperswarming adaptations in a bacterium improve collective motility without enhancing single cell motility. Soft Matter 10, 2405–2413 (2014). 24622509 10.1039/c3sm53127a PMC3955847

[b32] Sugiyama S., Cragoe E. J. & Imae Y. Amiloride, a specific inhibitor for the Na^+^-driven flagellar motors of alkalophilic *Bacillus*. J. Biol. Chem. 263, 8215–8219 (1988). 3372520

[b33] Spengler G. . Inhibitory action of a new proton pump inhibitor, trifluoromethyl ketone derivative, against the motility of clarithromycin-susceptible and-resistant *Helicobacter pylori*. Int. J. Antimicrob. Ag. 23, 631–633 (2004). 10.1016/j.ijantimicag.2003.11.010 15194136

[b34] Rasmussen L. . A high-throughput screening assay for inhibitors of bacterial motility identifies a novel inhibitor of the Na^+^-driven flagellar motor and virulence gene expression in *Vibrio cholerae*. Antimicrob. Agents Ch. 55, 4134–4143 (2011). 10.1128/AAC.00482-11 PMC3165335 21709090

[b35] Nishiyama M. & Sowa Y. Microscopic analysis of bacterial motility at high pressure. Biophys. J. 102, 1872–1880 (2012). 22768943 10.1016/j.bpj.2012.03.033 PMC3328721

[b36] Belas R. When the swimming gets tough, the tough form a biofilm. Mol. Microbiol. 90, 1–5 (2013). 23927648 10.1111/mmi.12354

[b37] Cairns L. S., Marlow V. L., Bissett E., Ostrowski A. & Stanley-Wall N. R. A mechanical signal transmitted by the flagellum controls signalling in *Bacillus subtilis*. Mol. Microbiol. 90, 6–21 (2013). 23888912 10.1111/mmi.12342 PMC3963450

[b38] Harwood C. S., Fosnaugh K. & Dispensa M. Flagellation of *Pseudomonas putida* and analysis of its motile behavior. J. Bacteriol. 171, 4063–4066 (1989). 2738028 10.1128/jb.171.7.4063-4066.1989 PMC210162

[b39] van Elsas J. D., Semenov A. V., Costa R. & Trevors J. T. Survival of *Escherichia coli* in the environment: fundamental and public health aspects. ISME J. 5, 173–183 (2011). 20574458 10.1038/ismej.2010.80 PMC3105702

[b40] Wong P. T. W. & Griffin D. M. Bacterial movement at high matric potentials—I. In artificial and natural soils. Soil Biol. Biochem. 8, 215–218 (1976).

[b41] Bashan Y. & Levanony H. Horizontal and vertical movement of *Azospirillum brasilense* Cd in the soil and along the rhizosphere of wheat and weeds in controlled and field environments. Microbiology 133, 3473–3480 (1987).

[b42] Berg H. C. E. coli in Motion (Springer, 2004).

[b43] Young I. M. & Crawford J. W. Interactions and self-organization in the soil-microbe complex. Science 304, 1634–1637 (2004). 15192219 10.1126/science.1097394

[b44] Blattner F. R. . The complete genome sequence of *Escherichia coli* K-12. Science 277, 1453–1462 (1997). 9278503 10.1126/science.277.5331.1453

[b45] Sambrook J. & Russel D. W. Molecular Cloning, A Laboratory Manual 3rd edn (Cold Spring Harbor Laboratory Press, 2001).

[b46] Hillel D. Introduction to Environmental Soil Physics (Academic press, 2003).

