# Supplementary material for: Bacterial flagellar motility on hydrated rough surfaces controlled by aqueous film thickness and connectedness
Source: Sci Rep. 2016 Jan 13;6:19409. doi: 10.1038/srep19409 (PMC4725831; doi:10.1038/srep19409)
Supplement: Supplementary Information [file srep19409-s1.pdf]

Title: Bacterial flagellar motility on hydrated rough surfaces controlled by aqueous film thickness and connectedness

Authors: Robin Tecon, Dani Or

Soil & Terrestrial Environmental Physics, Department of Environmental Systems Science, ETH Zürich, Universitätstrasse 16, 8092 Zürich, Switzerland.

Supplementary Information

Suppl. Figure S1: Aqueous film thickness and connectedness on rough surfaces and their effects on bacterial cell velocity and dispersal (p. 2)

Suppl. Figure S2: The ceramic Porous Surface Model (PSM) (p. 3)

Suppl. Figure S3: Quantification of *P. protegens* flagellar motility in aqueous films and comparison with model simulations (p. 4)

Suppl. Figure S4: Distribution of *P. protegens* swimming velocities (p. 5)

Suppl. Figure S5: *Escherichia coli* flagellar motility in aqueous films (p. 6)

Suppl. Video S1-S3 legends (p. 7)

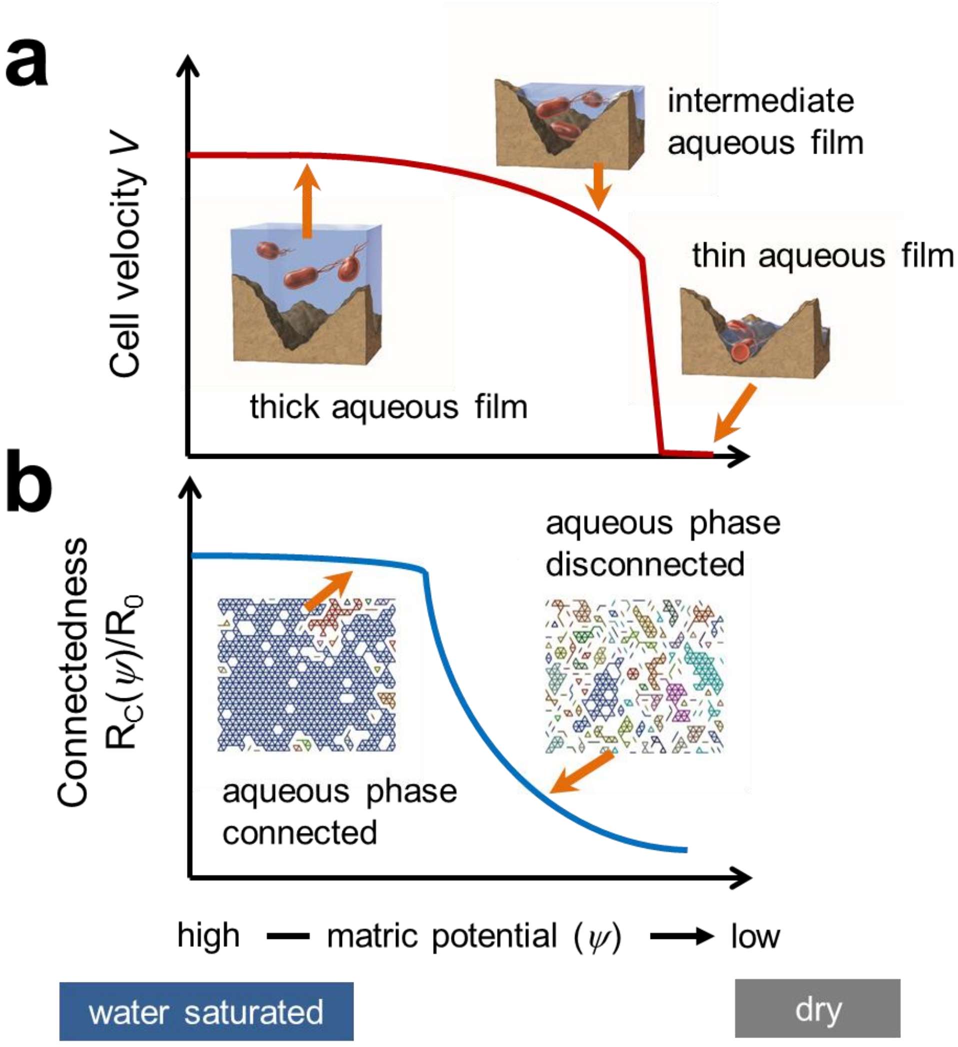

Supplementary Figure S1. Aqueous film thickness and connectedness on rough surfaces and their effects on bacterial cell velocity and dispersal. The figure illustrates changes in swimming cell velocity (**a**) and aqueous film connectivity (i.e., connectedness) (**b**) as function of matric potential (ψ) on hydrated rough surfaces. Drier conditions (low matric potential) influence bacterial flagellar motility primarily in two ways. First, thin aqueous films increase viscous and capillary pinning forces acting on individual swimming cells. These forces reduce cell velocity V and bring it to zero when the aqueous film is thinner than a cell's diameter (**a**). Second, drier conditions create disconnected microhabitats for bacteria, as very thin aqueous films cannot be crossed by swimming cells (**b**). This reduces the connectedness in the system, which is defined as the aqueous domain size at a given matric potential ψ (R_C) normalized by the maximal domain size (R_0) at system saturation (high matric potential, $\psi = 0$). In (**b**), colours indicate different isolated aqueous domains in an idealized hydrated rough surface at high (wet) or low (dry) matric potential.

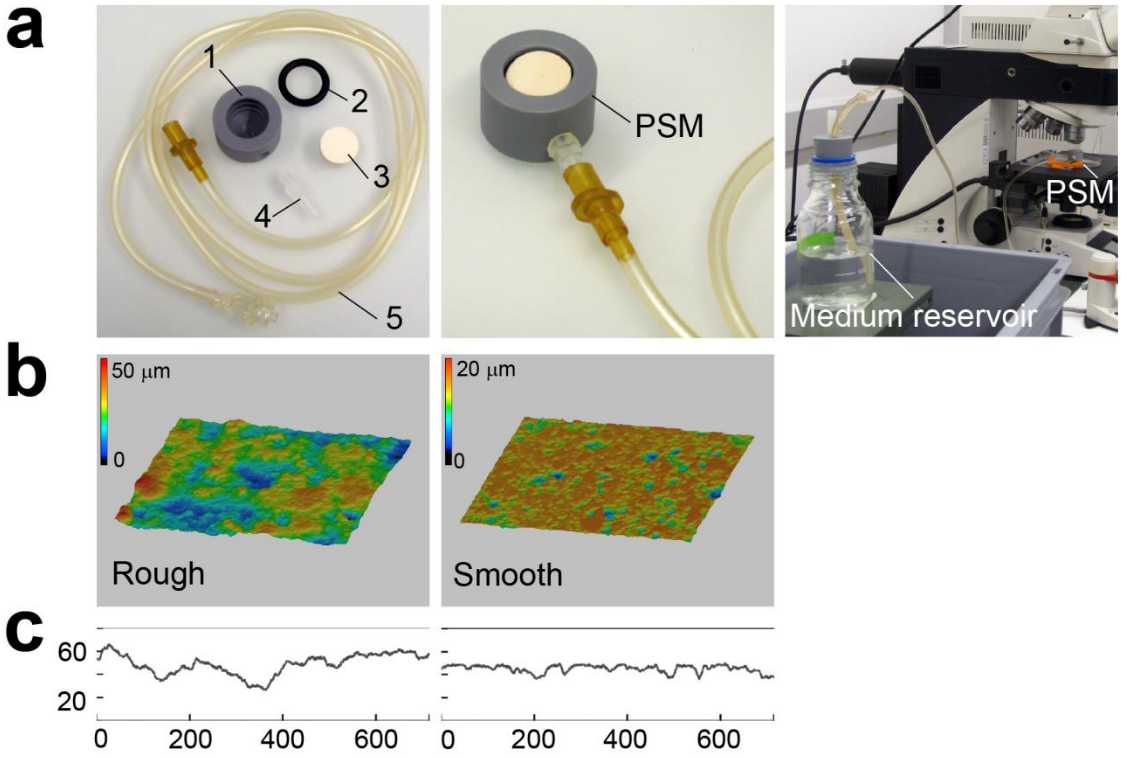

Supplementary Figure S2. The ceramic Porous Surface Model (PSM). (**a**) Assembly of a PSM system. PSM elements are: 1) PVC holder; 2) O-ring; 3) Ceramic disc (diameter 14 mm); 4) Connector; 5) Heidelberg extension line. PSM is installed on a microscope stage for direct surface observation. Hydration level on the PSM is controlled by a prescribed suction (equivalent to the effect of matric potential in porous media). To do so, the bottle containing the medium reservoir has to be placed at a lower level than the PSM. The height difference between the ceramic surface (PSM) and the liquid surface in the medium reservoir determines the resulting applied suction. The equivalent matric potential is given by the relation $\psi_m = \rho g h$, with ρ the density of water, g the acceleration of gravity and h the height of the liquid column. (**b**) 3D scanning laser micrographs of PSM with two different levels of roughness. The scanned surface is 500 µm x 715 µm. Roughness reduction is obtained by polishing the ceramic surface. Colour coding: red indicates high z position, blue low z position. (**c**) xz-cross sections showing surface profile of a mid-section transect of ‘rough’ and ‘smooth’ PSM (scales are in µm). The measured root mean square (RMS) roughness (in µm) was 10.3 ± 1.2 (SD) and 5.2 ± 1.2 (SD) for ‘rough’ and ‘smooth’ PSM, respectively.

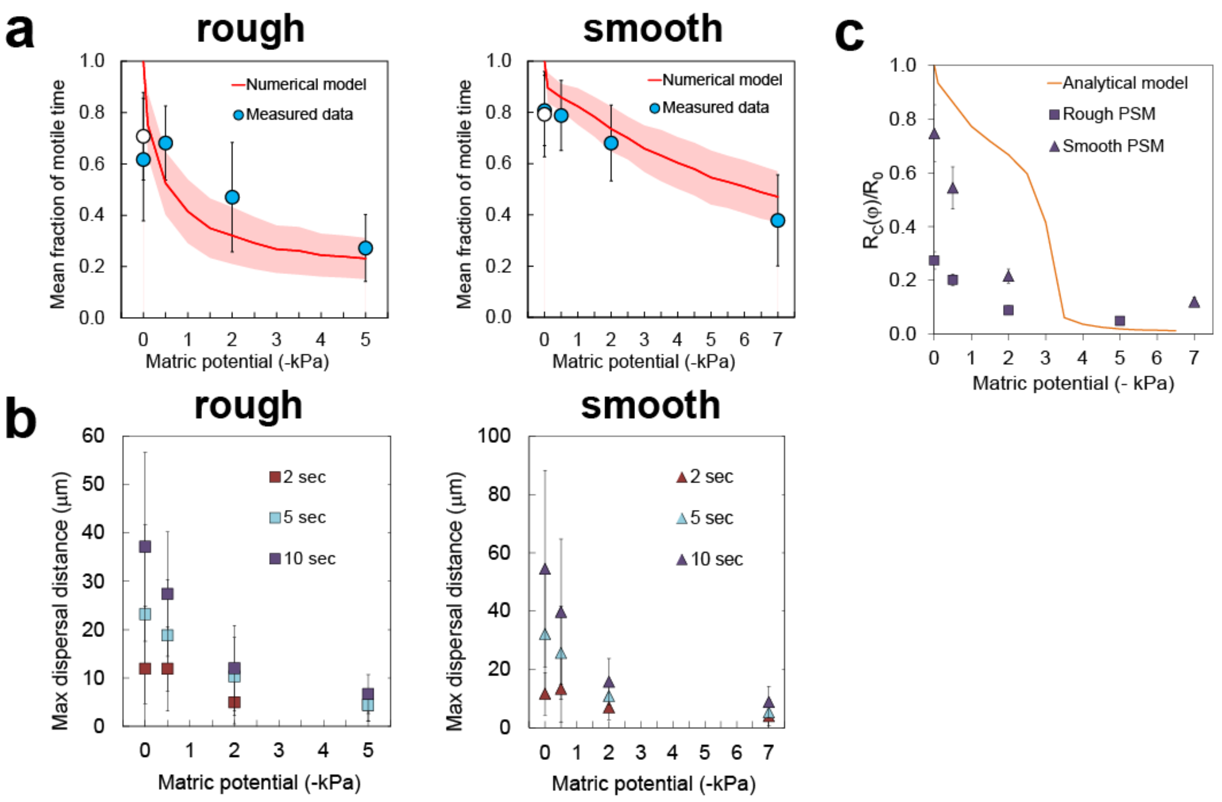

Supplementary Figure S3. Quantification of *P. protegens* flagellar motility in aqueous films and comparison with model simulations. This figure analyses the same swimming trajectories as in Fig. 5a,b. (**a**) Mean fraction of motile time, defined as the fraction of intermediary time slots in which swimming velocity was >3 µm/s. Error bars are 1 SD. In each experiment matric potential was sequentially lowered (0 kPa, –0.5 kPa, *etc.*) and finally brought back to 0 kPa (indicated by an open circle). With numerical simulations, shaded area is ±1 SD calculated from 100 realizations. (**b**) shows the effect of reducing the swimming time to 5-sec or 2-sec in the analysis of the maximal dispersal distance on 'rough' (left) and 'smooth' (right) PSM. Error bars are 1 SD. (**c**) Experimental and theoretical estimates of aqueous habitat fragmentation with matric potential. Experimental values (symbols) obtained by normalizing the maximal dispersal distance at each matric potential by the maximal dispersal distance observed on a control glass slide, the line is the universal percolation theory prediction scaled by system length. Error bars are 1 standard error of the mean. The effective size of the largest cluster is predicted by percolation theory and expressed as $R_C(\psi) = R_0\ (\frac{N_C(\psi)}{N_0})^{1/\chi}$, where R_0 is the radius of the network size, N_C is the number of channels in the largest cluster, N_0 is the total number of channels, and χ characterizes the dimensionality of the network (with value of 91/48 for 2D systems). We used N_0 = 20,000 in our analytical model to scale the domain (results are insensitive to larger values).

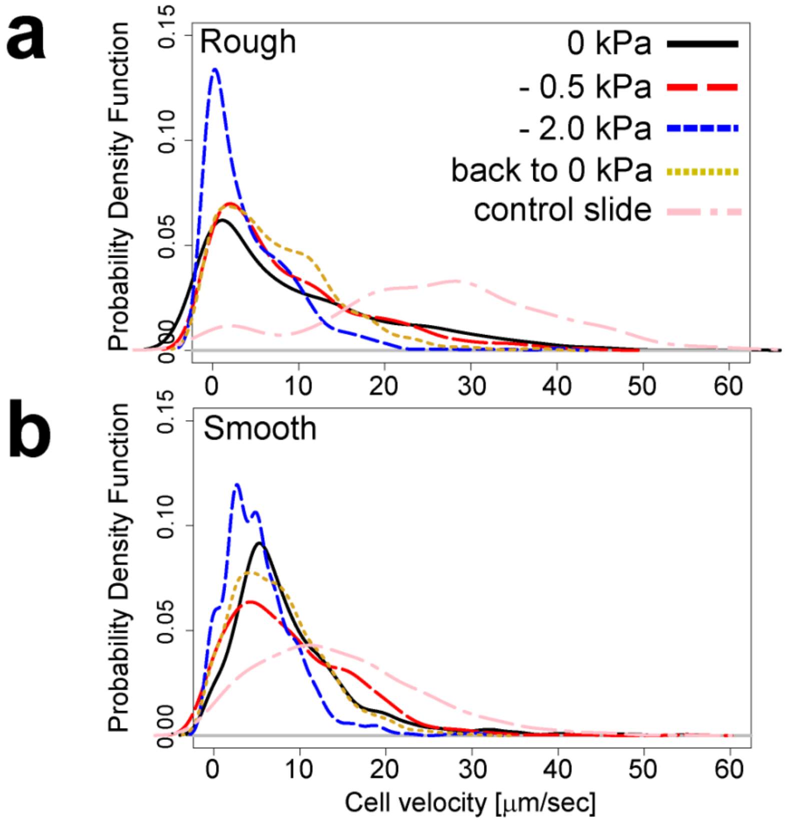

Supplementary Figure S4. Distribution of *P. protegens* swimming velocities. Individual cells velocities were measured on 'rough' (**a**) and 'smooth' (**b**) PSM. The data used to produce the probability density function of cell velocity is the same as presented in Fig. 5a,b.

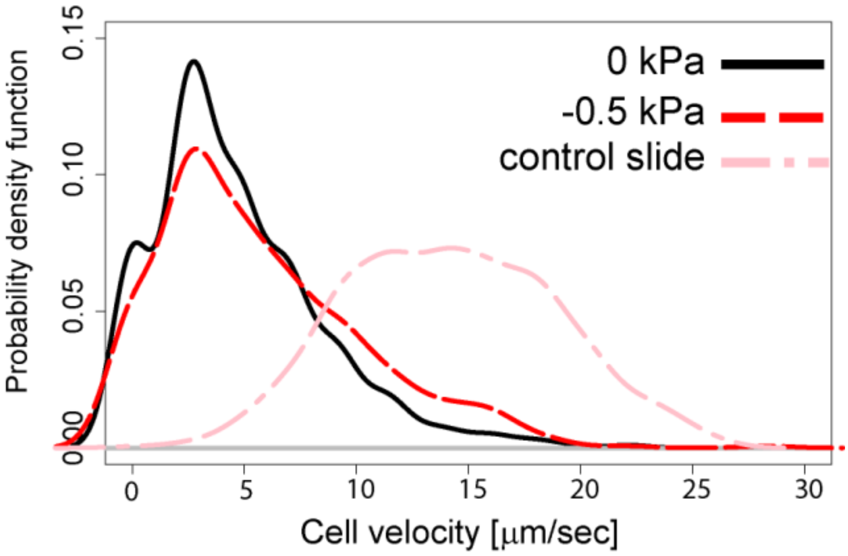

Supplementary Figure S5. *Escherichia coli* flagellar motility in aqueous films. Bacteria stained with Syto9 were visualized on 'rough' PSM by epifluorescence microscopy. Matric potential was sequentially lowered and finally brought back to 0, followed by a control of swimming between glass slides. We analyzed 13, 14 and 20 independent trajectories for 0 kPa, –0.5 kPa and control slide, respectively.

Supplementary Video S1. Real-time dynamics of aqueous film configuration during a drainage/rewetting sequence. The PSM surface was observed with 3D laser scanning microscopy. Water films appear as lighter area. At the beginning of the time-lapse movie matric potential is set at 0 kPa. At 10 seconds in the movie it is lowered to –10 kPa, which provokes rapid drainage. It is set back to 0 kPa at 30 seconds, leading to slower rewetting that brings back the initial water configuration. Red bar is 50 μm.

Supplementary Video S2. Time-lapse video of *Pseudomonas protegens* flagellar motility on 'smooth' PSM with matric potential set to 0 kPa. The PSM surface was observed with an epifluorescence microscope and a 10X objective, while bacteria were stained with the fluorescent dye Syto9. (Observation: 10 s, 36 pictures.) These conditions favour the dispersal of swimming cells.

Supplementary Video S3. Time-lapse video of *Pseudomonas protegens* flagellar motility on 'smooth' PSM with matric potential set to –2 kPa. The PSM surface was observed with an epifluorescence microscope and a 10X objective, while bacteria were stained with the fluorescent dye Syto9. (Observation: 10 s, 36 pictures.) These conditions strongly constrain the dispersal of swimming cells.
